# A novel rat model of tibial fracture for trauma researches: a combination of different types of fractures and soft tissue injuries

**DOI:** 10.1186/s13018-019-1386-4

**Published:** 2019-10-24

**Authors:** Enxian Shi, Gang Chen, Bengang Qin, Yi Yang, Jintao Fang, Liang Li, Yuanyuan Wang, Menghai Zhu, Jiantao Yang, Liqiang Gu

**Affiliations:** Department of Microsurgery & Orthopedic Trauma, The First Affiliated Hospital of Sun Yat-sen University, 58 Zhongshan 2nd Road, Guangzhou, 510080 Guangdong People’s Republic of China

**Keywords:** Fracture, Soft tissue injury, Model, Trauma, μCT

## Abstract

**Background:**

The outcomes for open tibial fractures with severe soft tissue injury are still a great challenge for all the trauma surgeons in the treatment. However, most of the existing open tibial fracture models can only provide minimal soft tissue injury which cannot meet the requirement of severe trauma research. Our goal is to investigate a novel tibial fracture model providing different fractures combined with soft tissue injury for better application in trauma research.

**Methods:**

A total of 144 Sprague-Dawley rats were randomly divided into 4 groups. With group 1 as control, the other groups sustained different right tibial fractures by the apparatus with buffer disc settings either 3 mm, 10 mm, or 15 mm. X-ray and computed tomography angiography (CTA) were performed at 6 h to evaluate the fracture patterns and vascular injuries. Peripheral blood and tibialis anterior muscle were harvested at 6 h, 1 day, 3 days, 7 days, 14 days, and 28 days for ELISA and histological analysis.

**Results:**

X-ray and μCT results indicated that different fractures combined with soft tissue injuries could be successfully provided in this model. According to OTA and Gustilo classification, the fractures and soft tissue injuries were evaluated and defined: 36 type I in group 2, 34 type II in group 3, and 36 type III in group 4. The CTA confirmed no arterial injuries in groups 1 and 2, 2 arterial injuries in group 3, and 35 in group 4. ELISA indicated that the levels of pro-inflammatory cytokines TNF-α and IL-1β were significantly higher in group 4 than in other groups, and the levels of anti-inflammatory cytokines TGF-β and IL-10 were significantly higher in surgery groups than in group 1 in later stage or throughout the entire process. HE, Masson, and caspase-3 stains confirmed the most severe inflammatory cell infiltration and apoptosis in group 4 which lasted longer than that in groups 2 and 3.

**Conclusions:**

The novel apparatus was valuable in performing different fractures combined with soft tissue injuries in a rat tibial fracture model with high reproducibility and providing a new selection for trauma research in the future.

## Introduction

The incidence of open tibial fracture as a part of isolated injury or polytrauma is on the rise due to increase in the incidence of motor vehicle accidents [1, 2]. The outcomes for severe open fractures are still a great challenge for all the trauma surgeons in the treatment [3, 4]. Especially, the soft tissue injury in open fractures still leads to various complications, such as infection, muscle necrosis, ischemia, and even limb loss [5, 6]. And it is generally accepted that the outcomes of fractures depended on not only the fracture itself but also the combined soft tissue injury. Thus, it is very critical to develop an animal model for open fracture research with appropriate consideration of soft tissue injury in fractures.

In previous studies, it was shown that the closed fracture was made by an open osteotomy through elimination of the added variable of local wound healing. Since then, a more standard closed fracture was created by making the femur “prepinned” with an intramedullary wire and subsequently fractured with a blunt guillotine [7–9]. However, existing open tibial fracture models can only provide minimal soft tissue injury, which leaded to the limited application in basic researches. This kind of minimal soft tissue injury in the existing fracture models was made according to the anatomical characteristics step-by-step instead of being caused by high energy. These models which could not mimic the clinic fractures which always occurred with corresponding soft tissue injuries in seconds are less applicable to trauma researches. Naturally, we purpose that a more appropriate rat fracture model which can mimic different fractures combined with soft tissue injuries will be a valuable supplement to the existing fracture models for trauma researches.

To meet these requirements, we made a modification to the traditional methods and designed a simple and adjustable apparatus with the buffer disc settings which could change the crash time of the hammer onto the rat’s legs and ultimately change the energy transferred to the involved extremity. These buffer discs made it easy and repeatable to provide various typical tibial fractures combined with soft tissue injuries.

In this experimental study, we aimed to investigate a novel tibial fracture model providing different fractures combined with soft tissue injury for better application in trauma research.

## Materials and methods

### Apparatus

**Fig. 1:**
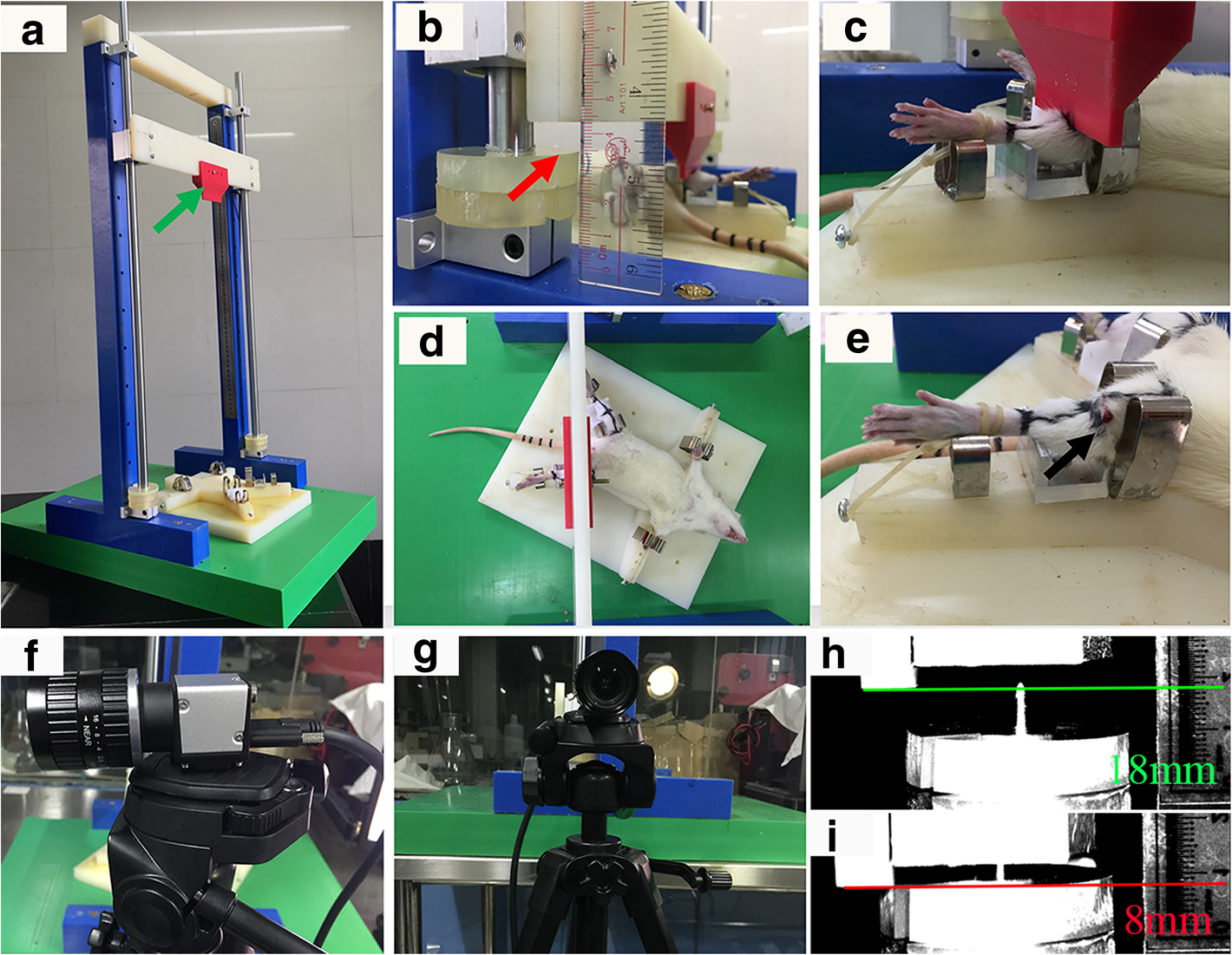
The fracture apparatus and the process of a typical moderate open fracture (type II). **a** The structure of the apparatus and the blade (green arrow) which was released from the top to provide a fracture. **b** The adjustable buffer disc system (red arrow) which was set to 10 mm. **c**, **d** The involved lower leg of a rat sustaining a crash injury by the blade. **e** A mild wound (black arrow) in the proximal third of the left tibia. **f**, **g** The anterior and lateral views of the high-speed camera system. **h** The moment when the blade began to crash on the leg of rat at the level of green line. **i** The moment when the blade stopped the crash on the leg of rat at the level of the red line, and the crash range was defined as the interval between the red and green lines (10 mm)

A modified apparatus was used in this model. The illustration of the apparatus was presented in Fig. 1 which consists of 5 parts: (a) a nylon frame, (b) a sliding plate with an adjustable blade (total weight 500 g), (c) a linear bearing system, (d) an adjustable polyurethane buffer disc setting, and (e) an operation panel with clamps. The adjustable buffer disc setting can be manipulated easily before operation to change crash range which was confirmed by a high-speed photography system (YVISON ltd, Shenzhen, China) (Fig. 1).

### Experimental procedure

A total of 144 S-D rats weighing from 200 to 250 g were used in this investigation. All animals were purchased from the Central of Experimental Animals of the Sun Yet-Sen University. The experimental design and procedures were approved by the Animal Care and Use Committee of the First Affiliated Hospital of Sun Yet-Sen University.

Rats were randomly divided into 4 groups (*n* = 36). Rats in group 1 served as control which only received anesthesia, while the other groups sustained fractures on the proximal third of the diaphysis in the right tibia by the apparatus. In groups 2, 3 and 4, the 500-g blade was released from the height of 50 cm as described previously [7]. The buffer disc setting was defined as the interval between the buffer disc and the frame when the blade was loaded on the involved extremity, and the settings of each group were different: 3 mm in group 2, 10 mm in group 3, and 15 mm in group 4. All procedures were conducted under general anesthesia with intraperitoneal injection of 40 mg/kg pentobarbital sodium. The right hind leg was shaved and disinfected in a sterile manner. Then, the rat was placed in the supine position, and the involved extremity was fixed on the operating panel (Fig. 1).

After performing X-ray and computed tomography angiography (CTA) at 6 h, the fracture was stabilized individually. Generally, the closed simple fracture is prone to be stabilized by Scotchcast casting tape (3 M, Inc., USA), while an open and comminuted fracture would be stabilized by a retrograde intramedullary Kirschner wire (0.8 mm) in a sterile manner after debridement and reduction [10]. Furthermore, the wire was to be cut and bent back onto itself before the incision was closed with absorbable sutures (Vicryl, Ethicon, Inc., USA).

All animals were kept on a heating pad, with the temperature at 37° during the recovery period. Systematic analgesia (buprenorphine at 0.01 mg/kg) was administered to erase the pain in all groups including group1 as control. Rats were given food and water ad libitum and monitored daily. All rats’ weight was monitored respectively before injury and 1 day, 3 days, 7 days, 14 days, and 28 days postoperatively.

### Radiology evaluation

To evaluate the fracture severity, anteroposterior (AP) radiographs of tibias were obtained at 6 h, 1 day, 3 days, 7 days, 14 days, and 28 days after fracture (Siemens, Germany). Additionally, CTA was performed to evaluate the combined vascular injury in the involved limb at 6 h postoperatively. The images were obtained after intravenous injection of Ultravist (centration of 300 mg/mL, Bayer HealthCare, Berlin, Germany) at the dose of 5 mg/kg/min through Inveon PET/CT (Siemens, Germany) with the scanning parameters as follows: 80 μm resolution, 80 KV voltage, and 500 μA current [11, 12].

### Injury assessment and categories

Based on the radiographical results and clinic characters, the ultimate fracture and soft tissue injury types were evaluated blindly by 2 senior orthopedic surgeons according to the concepts and principles of Gustilo and Orthopedic Trauma Association (OTA) classifications [13–15].

**Fig. 2:**
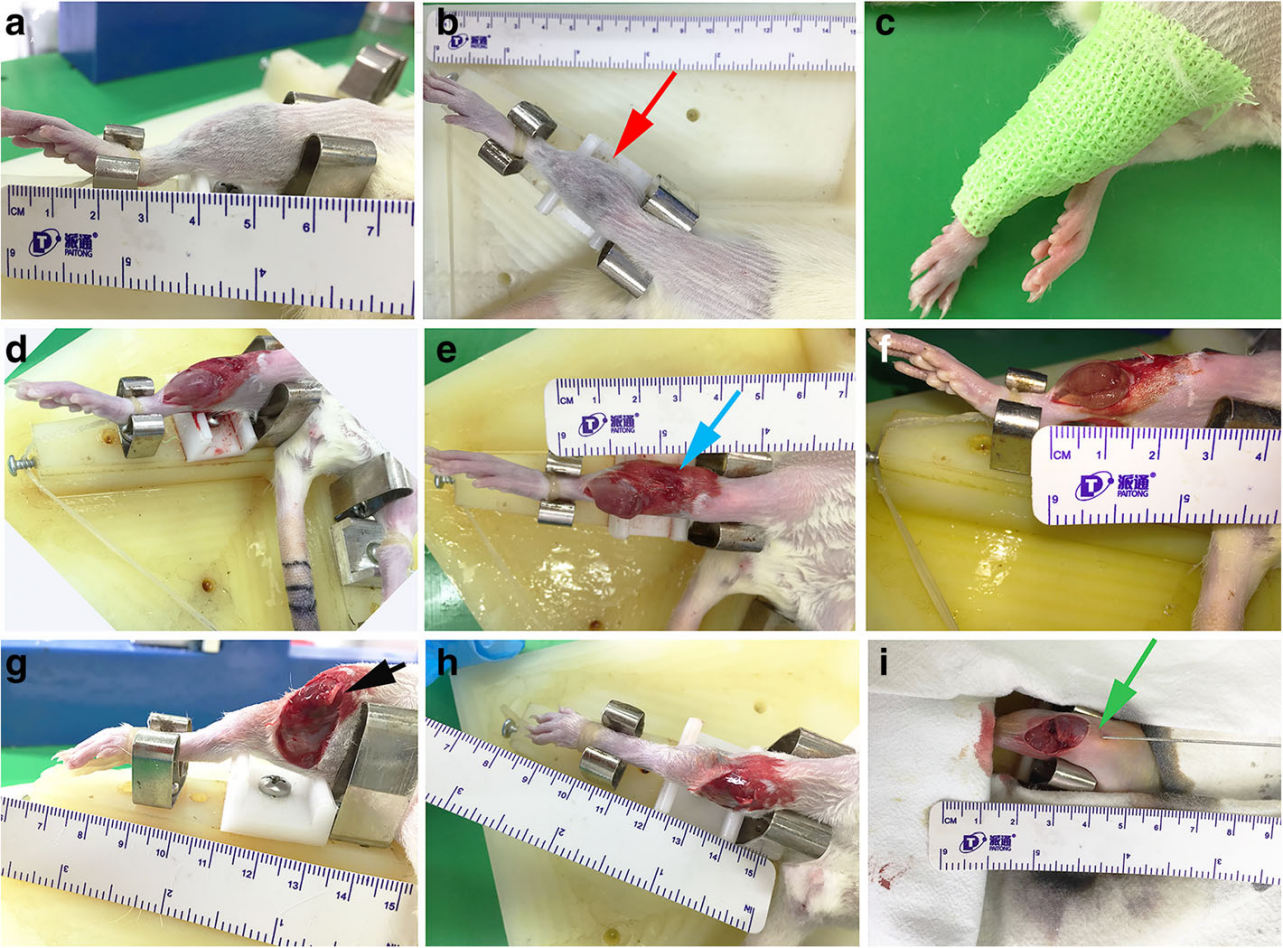
The process of three different types of fractures. **a**, **b** Lateral and anterior-posterior views of a closed fracture (type I) with a bruise on the skin of the fracture site (red arrow). **c** The cast used for immobilization after fracture. **d**–**f** Moderate skin lacerations and localized muscle injury in a type II fracture. **g**, **h** The severe wound and comminuted fracture with exfoliation of the periosteum and bone loss (black arrows). **i** The reduction and fixation of a type III fracture with a reversely implanted 0.8-mm intramedullary Kirshner wire

The fractures were divided into 3 categories in this study as below: type I fractures are simple closed fractures with minimal soft tissue injury which means only inside-out skin lesion and no muscle rupture or vascular injuries (Fig. 2); type II fractures are open and moderate comminuted fractures with moderate soft tissue injury which means skin lacerations, local muscle injury (laceration or slight rupture), and sometimes mild vascular lesion; and type III fractures are more severe comminuted fractures with extensive lacerations, severe muscle avulsion, and vascular injuries even ischemia in the distal extremity.

### Histology and ELISA examination

The blood and muscle were harvested to evaluate the systemic and local inflammation. These samples were collected at 1 day, 3 days, 7 days, 14 days, and 28 days postoperatively, with 6 rats sacrificed (via CO2 asphyxiation) in each group per time point. All samples were quantified in duplicate. Cytokine levels (TNF-α, IL-1β, TGF-β, and IL-10) in serum were tested by ELISA kits (CSB Ltd, USA) [16]. After perfusion with saline followed by 4% PFA, the anterio-tibilis were harvested for HE stain, Masson stain, and IHC (caspase-3) stain as previously described [17]. Positively stained cells were counted with 3 consecutive observation fields per section.

### Statistical analysis

All data were presented as the means ± SD and were analyzed by ANOVA to evaluate the differences between groups by SPSS version 22.0 software (IBM Corp, NY, USA). A *P* value < 0.01 was considered statistically significant.

## Results

### Fracture and vascular injury evaluation

**Fig. 3:**
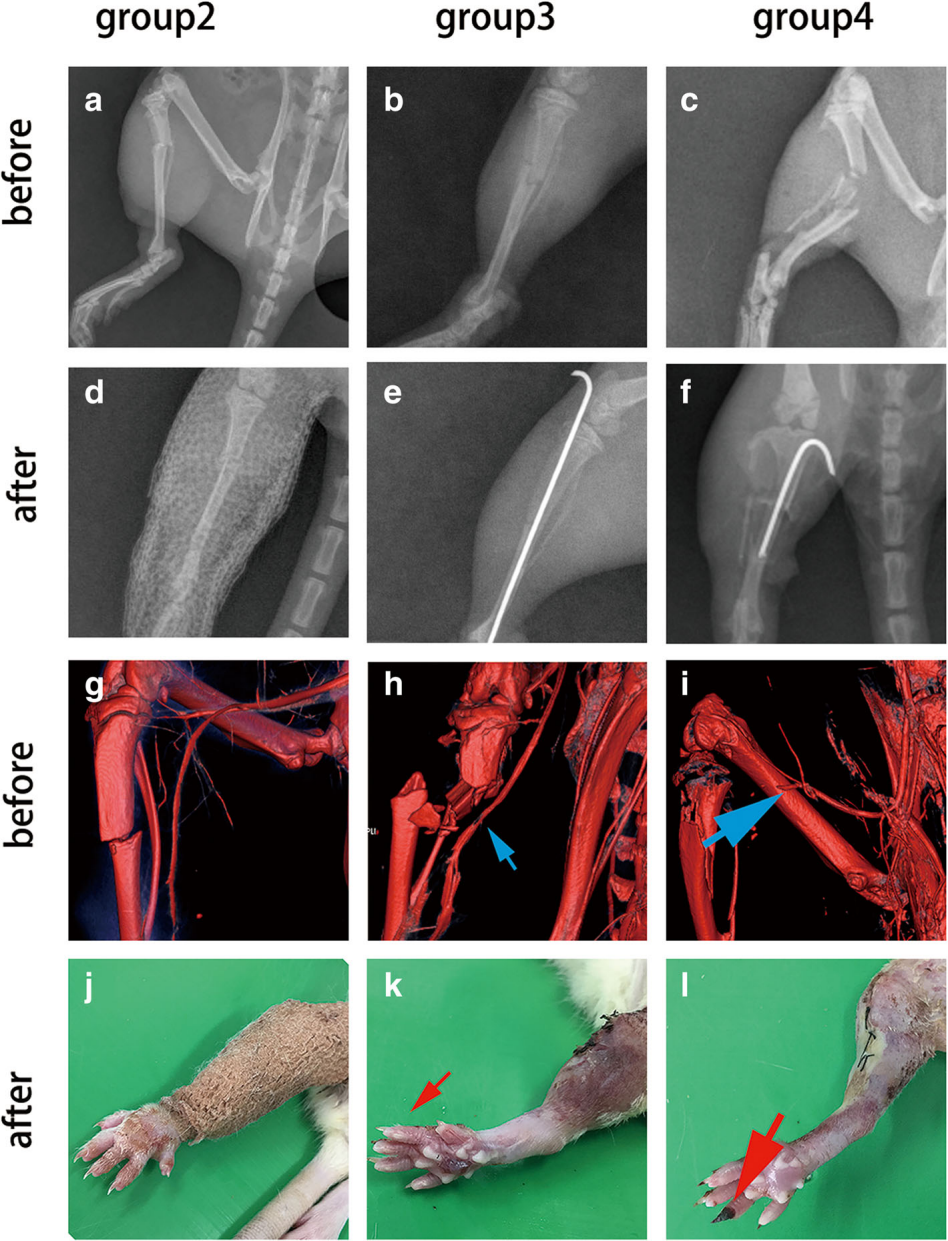
Radiology evaluation in different groups before and after fixation. **a** A simple transverse fracture. **b** A moderate comminuted fracture with small segments. **c** Severe comminuted fracture with large gone segments. **d** X-ray after plaster immobilization. **e**, **f** X-rays after intermedullary pin fixation. **g** CTA image without signs of vascular injury. **h** A moderate filling defect in the artery (small blue arrow). **i** Complete occlusion of the main artery of a lower extremity (large blue arrow). **j** Almost normal appearance of the distal part of the involved extremity after fixation. **k** Swollen toes without necrosis (small red arrow). **l** Partial necrosis of the toes (large red arrow)

The results of X-ray and μCT indicated that different fractures combined with soft tissue injuries were successfully provided by our apparatus (Fig. 3). The result of X-ray at 6 h showed that a simple transverse fracture happened in group 2, a moderate comminuted fracture with small segments happened in group 3, and a severe comminuted fracture with large gone segments occurred in group 4. The result of CTA showed no vascular injury in group 1 and group 2. Conversely, the vascular injury was significantly more severe in group 4 (36/36) compared to group 3 (2/36). In some cases of group 4, partial necrosis of toes was found at day 7 postoperative. However, no total necrosis or ischemia of the leg was found in any groups (Fig. 3). The fracture types and corresponding soft tissue injury distributions were described in Table 1. It was observed that no fractures occurred in group 1, while 36 type I fractures in group 2, 34 type II and 2 type I fractures in group 3, and 36 type III fractures in group 4.

**Table 1:** Category distributions of fracture and soft tissue injury in groups

	Type I^*^	Type II^*^	Type III^*^	Skin injury	Muscle injury	Bone injury	Vascular injury^#^
Group 1 (control)	0	0	0	0	0	0	0
Group 2 (BDS^※^ = 3 mm)	36	0	0	36+	0-	36+	0-
Group 3 (BDS^※^ = 10 mm)	0	34	2	34++	34++	34++	2+
Group 4 (BDS^※^ = 15 mm)	0	0	36	36+++	36+++	36+++	36++

*Type I fracture is a low-energy fracture (simple transverse fracture, like OTA type A) with minimal soft tissue damage which means only inside-out skin lesion and no muscle rupture or vascular injuries. Type II fracture is a medium-energy fracture with mild comminuted segments (like Gustilo type II) and skin lacerations, local muscle injury, and sometimes mild vascular lesion. Type III fracture is a high-energy fracture with significant comminuted segments (like Gustilo type III) and extensive lacerations, severe muscle avulsion, and vascular injuries even ischemia in the distal extremity

^#^The diameter of the vessel in injury site less than 50% of proximal vessel was defined as vascular injury in this study

^※^BDS (buffer disc settings) is defined as the interval between the buffer disc and the frame when the blade was loaded on the involved extremity

+, slight injury; ++, moderate injury; +++, severe injury

### Histology

**Fig. 4:**
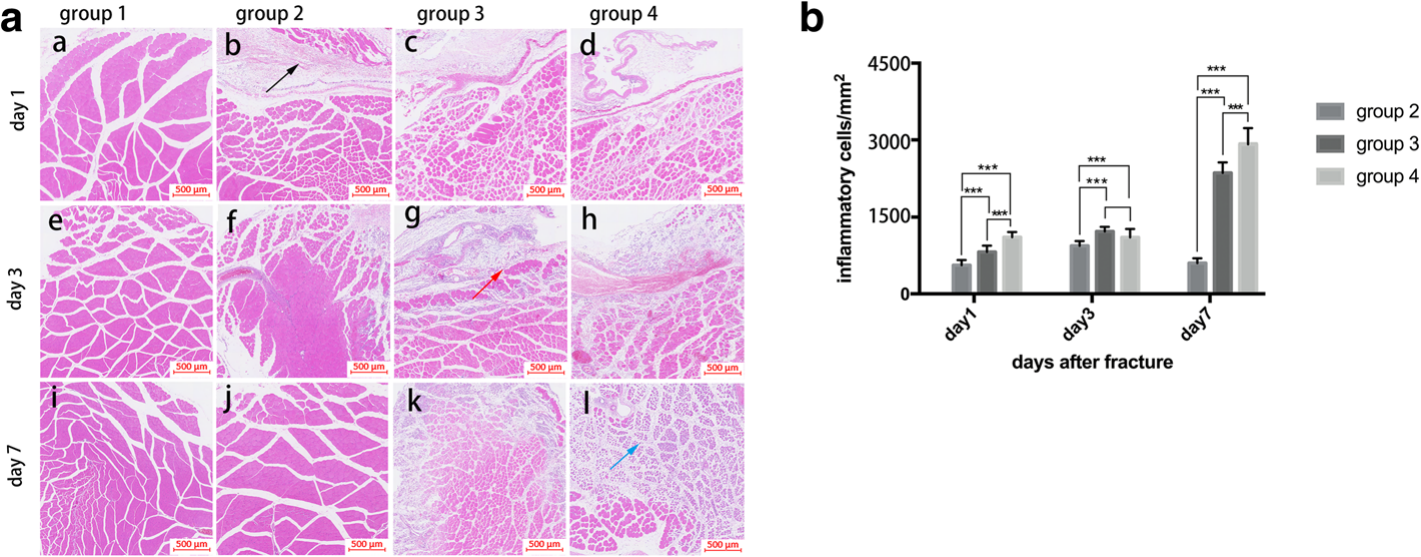
Inflammatory cell infiltration in the cross-sections of muscle adjacent to the fracture site in different groups. **a** (a, e, i) No obvious inflammatory cell infiltration in group 1, (b, f, j) mild infiltration (black arrow) at day 1 and obvious decline on day 3 and day 7 in group 2, (c, g, k) moderate infiltration (red arrow) lasting to day 3 and declining on day 7 in group 3, and (d, h, l) show severe inflammatory cell infiltration (blue arrow) lasting to the end of the first week in group 4. **b** The inflammatory cell counts are significantly more in groups 3 and 4 than in group 2 at day 1, day 3, and day 7 after fractures, and the most severe infiltration occurred in group 4 at day 1 and day 7. *P < 0.05; ***P < 0.01; scale bar = 500 μm

**Fig. 5:**
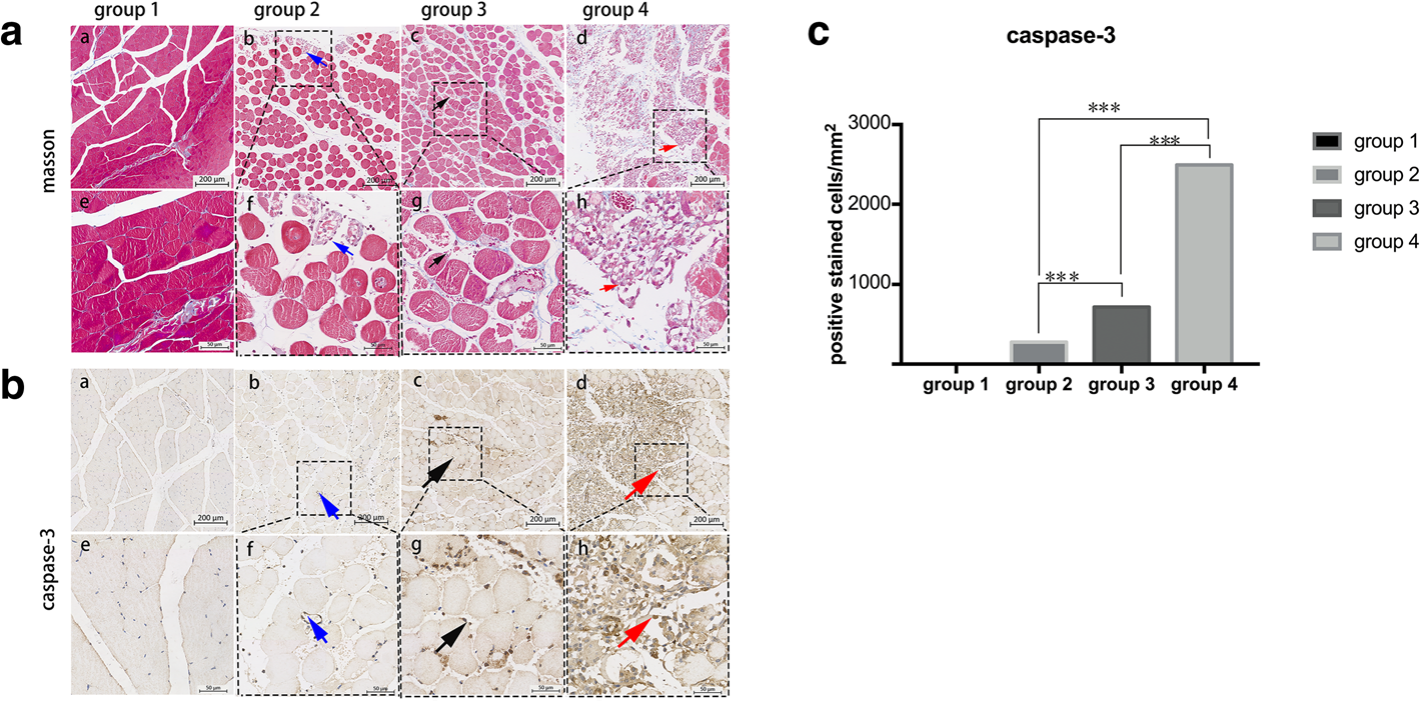
Masson and IHC stains in the muscle adjacent to the fracture site in different groups. **a** Masson staining results: (a, e) no obvious new collagen deposition and myofiber necrosis in group 1, (b, f) a few myofiber necrosis (blue arrow) in group 2, (c, g) moderate myofiber necrosis (black arrow) in group 3, and (d, h) severe myofiber necrosis (red arrow) in group 4. **b** Caspase-3 staining results: (a, e) no obvious necrotic inflammatory cells in group 1, (b, f) moderate necrosis with a few positively stained nuclei (blue arrow) in group 2, (c, g) obvious necrotic with numerous positively stained nuclei (black arrow) in group 3, and (d, h) severe necrotic myofibers with more positively stained nuclei (red arrow) in group 4. **c** Different caspase-3 positively stained cell distributions in different groups; it is the highest in group 4 than other groups. ***P < 0.01; scale bar = 200 μm in **a**; scale bar = 50 μm in **b**

The inflammatory cell infiltration in the cross-sections of muscle adjacent to the fracture site in different groups is presented in Fig. 4 and Fig. 5. HE-stained sections (Fig. 4) showed different characteristics of inflammatory cell infiltration among groups: no inflammatory cell infiltration was found in group 1, only mild inflammatory cell infiltration was found on day 1 in group 2, obvious infiltration continued from day 1 to day 3 and declined until day 7 in group 3, and severe inflammatory cell infiltration was found from day 1 to day 7 in group 4. Additionally, Masson staining of the muscles in each group on day 7 showed no obvious new collagen deposition and myofiber necrosis in group 1, a few myofiber necrosis in group 2, moderate myofiber necrosis in group 3, and severe myofiber necrosis in group 4 (Fig. 5).

HE stains and caspase-3 stains (Fig. 5) confirmed the most severe inflammatory cell infiltration and apoptosis in group 4 which lasted longer than that in groups 2 and 3. Quantificationally, the infiltration was the most severe in group 4 (2922 ± 132 cells/mm^2^), compared with group 3 (2349 ± 105 cells/mm^2^) and group 2 (783 ± 201 cells/mm^2^). The caspase-3-stained cells showed similar tendency between groups: 330 ± 55 cells/mm^2^ in group 2, 858 ± 49 cells/mm^2^ in group 3, and 2991 ± 127 cells/mm^2^ in group 4.

### Inflammatory response

**Fig. 6:**
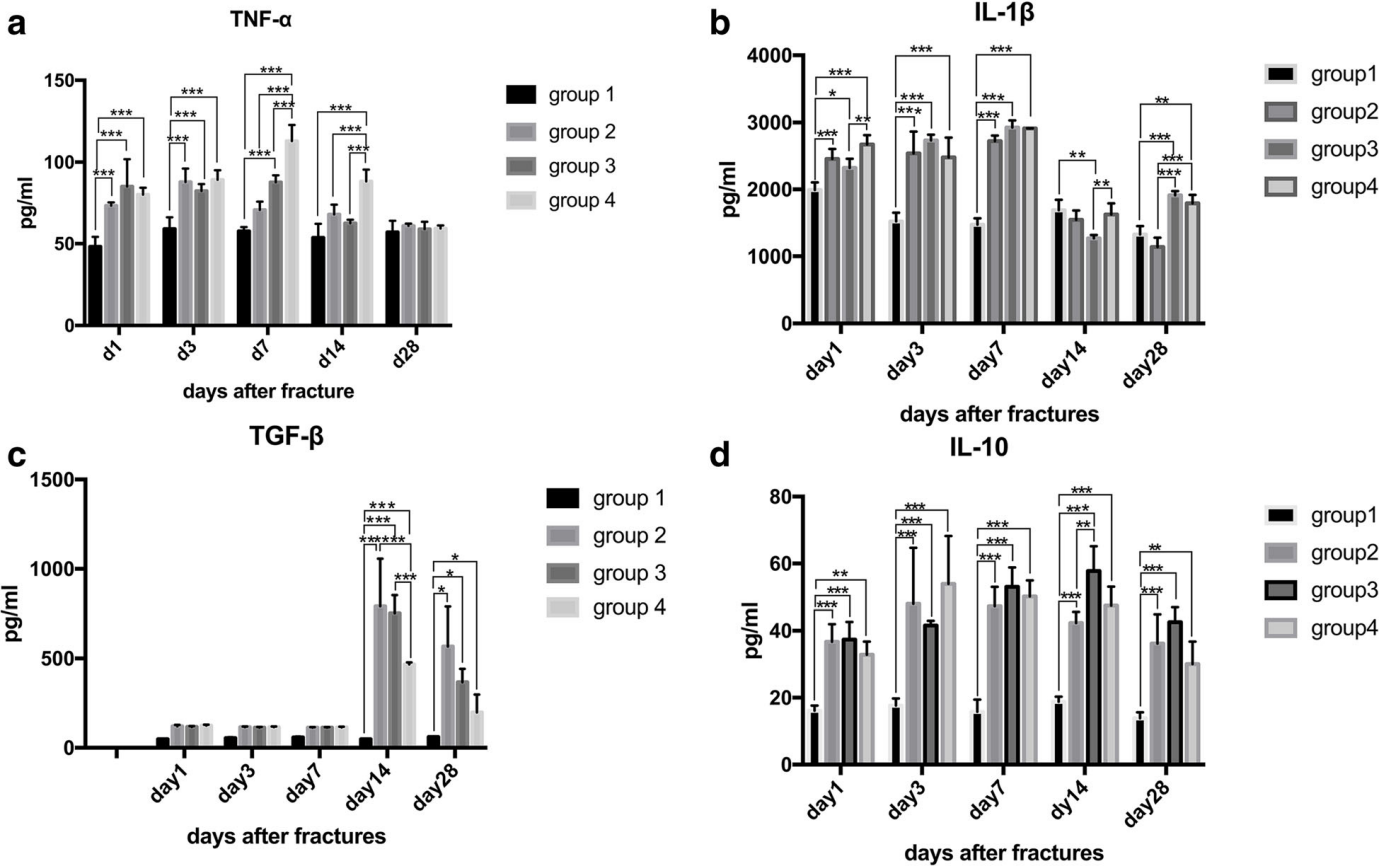
Cytokine levels in the serum from different groups after fracture. **a** The serum levels of TNF-α were significantly higher at day 1 in groups 2–4 than in group 1, and all decreased at day 7. **b** IL-1β levels were significantly higher in groups 2, 3, and 4 than in group 1 at day 1, day 3, and day 7, and all decreased after day 14. **c** TGF-β levels were similar between groups in the first week but were significantly higher in groups 2–4 than in group 1 at day 14 and day 28. **d** IL-10 levels were significantly higher in groups 2–4 than in group 1 throughout the entire process. *P < 0.05; ***P < 0.01

The pro-inflammatory cytokines (TNF-α and IL-1β) and the anti-inflammatory cytokines (TGF-β and IL-10) were detected by ELISA kits in different groups (Fig. 6). Compared with group 1 (control group), the pro-inflammatory cytokines remarkably increased in surgery groups and the levels of TNF-α and IL-1β were significantly higher in group 4 than in other groups (Fig. 6). The serum level of TNF-α and IL-1β kept in a high level from day 1 to day 7 and started to decrease on day 14 in surgery groups. Additionally, the corresponding duration of these pro-inflammatory cytokines was the longest in group 4. The ELISA results of the anti-inflammatory cytokines showed that TGF-β levels were similar between groups in the first week but were significantly higher in surgery groups than in group 1 from day 14 to day 28, while IL-10 levels were significantly higher in surgery groups than in group 1 throughout the entire process.

## Discussion

Open fractures have drawn considerable attentions recently [6]. Fractures of the tibial diaphysis are the most common long bone fracture, and approximately 24% of these fractures are open [18]. The soft tissue injury is one of the most complex problems due to its diversity in diagnosis and management of open fractures [19]. The high-energy nature of most of these fractures contributes to the increased proportion of Gustilo type III. In the epidemiologic study, Court-Brown et al. found that nearly 60% of open tibial shaft fractures were Gustilo type III [20]. Thus, a more appropriate rat fracture model which could mimic different fractures combined with soft tissue injuries will be valuable in trauma researches.

There are several different open fracture rat models modified from the classical model performing transverse osteotomy combined with skin incision and resections of muscle around the fracture site [10, 21]. However, most models provided closed or open fractures with only minimal soft tissue injury which was not caused by high energy, and these models were made according to the anatomical characteristics step-by-step and could not mimic the clinic fractures which always occurred with corresponding soft tissue injuries in seconds. But the pathological changes in an open fracture are too complicated to be mimicked by this simple method because soft tissues consist of several complex structures, such as skin, muscles, and vessels. This main flaw leaded to the limited application of these models in trauma researches. So compared with these existing models, our novel approach would have been more impactful in trauma research because we could perform different fractures combined with soft tissue injuries in a rat tibial fracture model with high reproducibility.

In our experiment, we made a modification and designed a simple and adjustable apparatus with buffer disc settings. A high-speed photography system was used in the pre-experiment to explore the appropriate buffer disc setting parameters. We tried different buffer disc setting parameters including 1 mm to 15 mm, evaluated the fracture types by X-ray immediately, and assessed the associated soft tissue injury by two senior orthopedic surgeons. The results indicated that when the buffer disc setting was settled at 3 mm, the most common occurred fractures would be closed fractures with minimal soft tissue injury. And more severe fractures with moderate soft tissue injury would be expected at 10 mm. Furthermore, the most severe fracture combined with extensive lacerations, severe muscle avulsion, and even vascular injuries would be expected at 15 mm.

After analyzing the result of pre-experiment comprehensively, the 3 mm, 10 mm, and 15 mm were purposed to provide three types of fractures combined with soft tissue injuries based on concepts of Gustilo classification [13–15]. Type I are simple closed fractures with only mild ecchymosis, type II are open and moderate comminuted fractures with skin laceration and slight muscle rupture but without obvious ischemia, and type III are more severe comminuted fractures with segmental bone loss and severe muscle avulsion and ischemia in the distal extremity [14, 15]. The types of fractures combined with soft tissue injuries in our study were evaluated by this modified fracture categories.

We evaluated the fracture patterns by obtaining radiographic image of rats’ involved limbs at 6 h after fracture. The results of X-ray and μCT indicated that different fractures combined with soft tissue injuries were successfully provided by our apparatus. The anteroposterior (AP) radiographs of tibias showed that a simple transverse fracture happened in group 2, a moderate comminuted fracture with small segments happened in group 3, and a severe comminuted fracture with large gone segments occurred in group 4 (Fig. 3). The fracture distribution showed no fractures occurred in group 1, while 36 type I fractures in group 2, 34 type II and 2 type I fractures in group 3, and 36 type III fractures in group 4(Table 1). These results confirmed that fracture distributions differed between groups which kept in line with different buffer disc settings and fracture categories.

Vascular injury was another predominate factor in outcomes of open fractures [22, 23]. However, it was rarely described in existing fracture models. CTA is a unique radiographic method that demonstrates the arterial vasculature structures through 3D volumetric reconstruction which could rapidly detect vascular injuries [24]. In our study, CTA results showed moderate and partial vascular injuries in group 3 (2/36) and obvious vascular injuries in group 4 (36/36) (Fig. 3). We contributed these results to the buffer disc settings which changed the crash range of the blade and subsequently changed the severity of injuries. What is more, rats in group 4 suffered obvious vascular injuries according to the result of CTA, and some of them had partial necrosis of toes at day 7 postoperative (Fig. 3). However, no total necrosis or ischemia of the leg was found in any groups. This suggested that in addition to fracture patterns, soft tissue injury may also have a profound effect on the outcome of trauma. It may be important to take soft tissue injury into account when we perform basic researches on trauma. Our novel model was more useful to meet these requirements and provided a new selection for trauma researches. And the results of weight monitoring showed that rats suffering the most severe fracture and soft tissue injury had the obvious malnutrition in group 4 (Additional file 1: Figure S1). As described in the previous study, there was a high prevalence of malnutrition among the trauma patients [25]. In our experiment, the characteristics of weight change in all groups showed that our model could effectively provide different fracture types which mimicked the clinical cases.

Recent investigations have demonstrated that inflammatory responses followed by infiltration of inflammatory cells and release of cytokines would occur after fractures [16]. In our study, histology and ELISA were performed to evaluate the inflammatory cell infiltration, necrosis, and serum cytokines after fracture. The results of HE stains, Masson stain, and caspase-3 demonstrated different levels and durations of infiltration between groups. Especially, the most severe inflammatory cell infiltration and the longest duration of infiltration occurred in group 4. These results indicated the severity and duration of inflammation depended on the severity of fractures which has been demonstrated in other investigations [26]. Additionally, ELISA analysis of inflammatory cytokines was performed to evaluate the systemic inflammation after fractures. Skeletal and tissue injuries may cause a hyper-inflammatory reaction of the immune system manifested by elevation in levels of pro-inflammatory cytokines, and the massive secretion of pro-inflammatory cytokines usually induces upregulation of anti-inflammatory cytokines such as IL-4 and regulatory cytokines such as transforming growth factor-beta (TGF-beta) and IL-10, which results in a decrease of the severity of the inflammatory reaction [27, 28]. In our study, compared with group 1 (control group), the levels of the pro-inflammatory cytokine TNF-α and IL-1β in groups 3 and 4 were significantly higher than those in groups 1 and 2. Additionally, the corresponding duration of these pro-inflammatory cytokines were the longest in group 4. The anti-inflammatory cytokine level in rats showed that TGF-β levels were significantly higher in surgery groups than in group 1 in the later stage, and IL-10 levels were significantly higher in surgery groups than in group 1 throughout the entire process. The increase in pro-inflammatory cytokines is evident soon after injuries (6 h) and lasts usually for 24–48 h in most cases, and the duration of this reaction depends on the severity of the trauma and the basic state of the patient [27, 28]. In our experiment, the inflammatory cytokines in rats’ blood samples showed the similar trend as that in most clinical cases. This inflammatory change also showed that we performed this novel tibial fracture model providing different fractures combined with soft tissue injury successfully.

There are several advantages in our model. Firstly, various fractures combined with soft tissue injuries could be successfully provided with indicated buffer disc settings. Our novel model could provide different soft tissue injuries which mimicked the clinical trauma cases and assess the severity of soft tissue injuries in multiple methods. Secondly, three fracture categories which derived from the concept of OTA and Gustilo classifications could be created in our model with high repeatability. These fracture types in our model would reflect a similar progression of severity as the Gustilo types, but not a radical copy of them. We contribute it to the design of the buffer disc settings which can be manipulated easily before operation and change the crash range of the blade onto the involved leg and lead to the indirect change of the fracture and soft tissue injury types. Additionally, to our knowledge, it is the first application of CTA in the evaluation of vascular injuries in open tibial fracture models. Furthermore, this novel model could be used in researches focused on the cross-talk between bone union and inflammatory microenvironments which could contribute to the nonunion of fractures.

There are several limitations of this study. First, the study is based on the short-term observation of fractures without long-term follow-up to assess bone union and functional recovery. Second, buffer disc setting parameters need more tests before its application in rats with different weight and diameter of the extremities or in other animals. What is more, vascular injuries in cases of open fracture are caused by external injury as well as internal injury due to fracture. But as it was difficult to perform a standardized and accurate assessment of internal injuries in a rat fracture model, we used CTA to assess different degrees of vascular injuries only caused by external injury in our experimental. Fourthly, body weight, blood loss, and fluids can have profound impacts both on injuries and inflammatory responses. In our experiment, we monitored the body weight preoperatively and postoperatively but missed monitoring and controlling the blood loss and fluids which were difficult to perform standardly in rats. Fifthly, wire fixation extending outside of the knee joint could have induced soft tissue injury itself. Although we harvested the tibialis anterior muscle near the fracture site for histological analysis, the wire may have an effect on levels of inflammatory cytokines in the peripheral blood. Additionally, we did not give any antibiotics to the rats after fractures. Antibiotics [29] given according to the bacteria in open fracture in clinic played a critical role in the inflammatory response.

## Conclusions

The novel apparatus was valuable in performing different fractures combined with soft tissue injuries in a rat tibial fracture model with high reproducibility. Therefore, this novel rat model of tibial fracture is effective and provides a new selection in trauma research.

## Supplementary information

**Additional file 1: Figure S1.** The change of rats’ body weight in groups during the whole process. The curve indicated that the body weight of rats significantly decreased in group 3 and group 4, and rats in group 4 had the most obvious loss of body weight. * *P* < 0.05; ** *P* < 0.01; ** * *P* < 0.00

## Data Availability

The datasets used and analyzed during the current study are available from the corresponding author on reasonable request, taking into account any confidentiality.

## Abbreviations

AP: Anteroposterior
CTA: Computed tomography angiography
ELISA: Enzyme-linked immunosorbent assay
IL-1β: Interleukin-1 beta
OTA: Orthopedic Trauma Association
S-D rats: Sprague-Dawley rats
TNF-α: Tumor necrosis factor α
μCT: Microcomputed tomography
