# Supplementary figures and images for: A novel rat model of tibial fracture for trauma researches: a combination of different types of fractures and soft tissue injuries
Source: J Orthop Surg Res. 2019 Oct 24;14:333. doi: 10.1186/s13018-019-1386-4 (PMC6813134; doi:10.1186/s13018-019-1386-4)

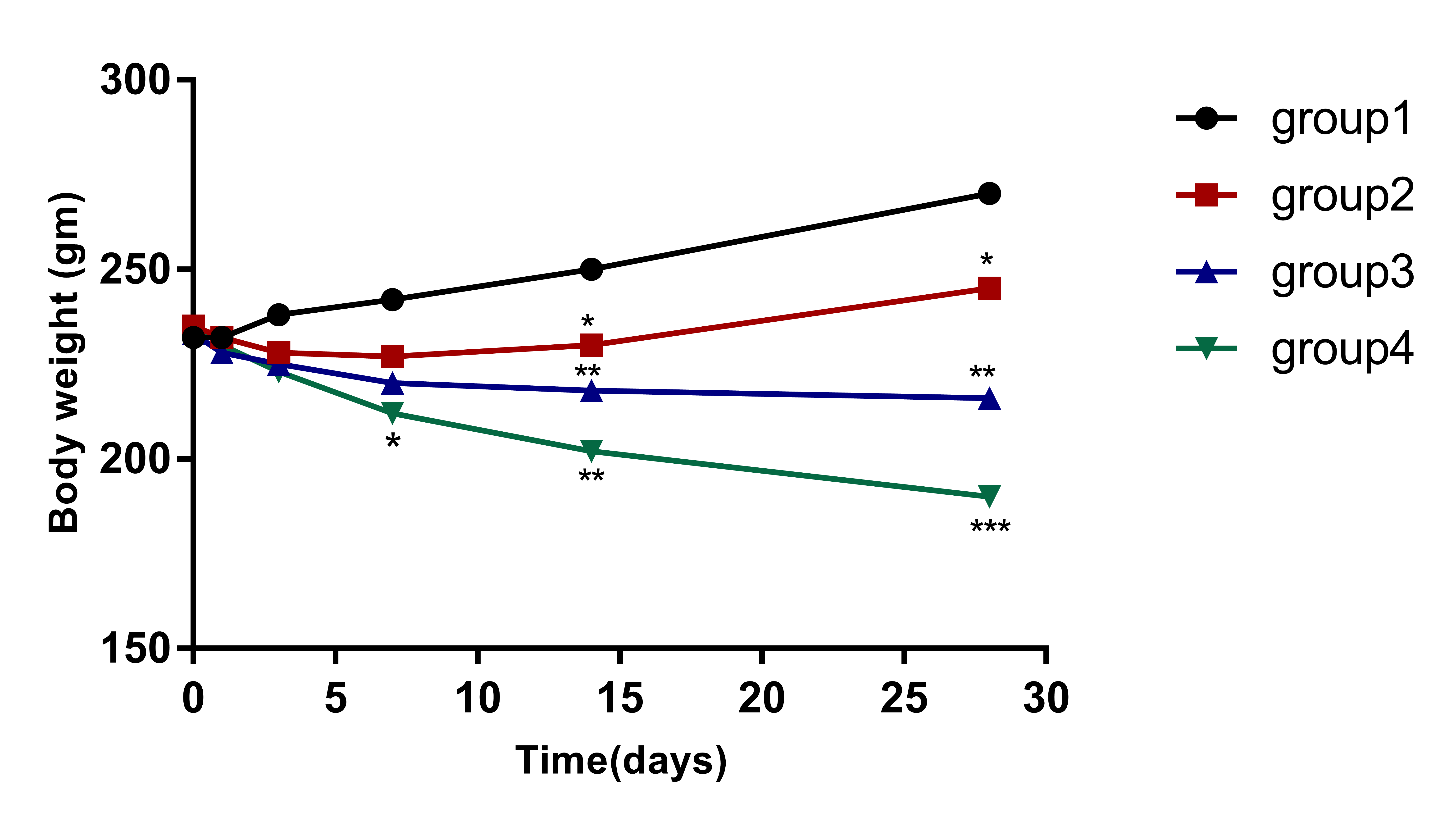

Supplement: Supplementary file 1 — Additional file 1: Figure S1. The change of rats’ body weight in groups during the whole process. The curve indicated that the body weight of rats significantly decreased in group 3 and group 4, and rats in group 4 had the most obvious loss of body weight. * P < 0.05; ** P < 0.01; ** * P < 0.00 [file 13018_2019_1386_MOESM1_ESM.tif]
